# Role of Amyloid Fibrils in Enhancing the Protective Properties of Microbial Melanin against Oxidative Stress, UV Damage, and AGE-Related Cytotoxicity in Human Keratinocytes

**DOI:** 10.1021/acsomega.6c05125

**Published:** 2026-08-10

**Authors:** Shana Perrella, Talayeh Kordjazi, Maria Liccardo, Loredana Mariniello, Ivana Sirangelo, Odile Francesca Restaino, Clara Iannuzzi

**Affiliations:** † Department of Precision Medicine, University of Campania “Luigi Vanvitelli”, Via L. De Crecchio 7, 80138 Naples, Italy; ‡ Department of Chemical Sciences, University of Naples Federico II, Monte Sant’Angelo Campus, Via Cintia 4, 80126 Naples, Italy

## Abstract

The biological properties of microbial melanins, whose structures resemble those of animal-derived eumelanin pigments, have been poorly investigated in mammalian cell systems, as well as the role of amyloid fibrils in modulating melanin activities. Indeed, although amyloid fibrils are known to be crucial in the melanogenesis process, their functional role is not fully understood. In this study, we have investigated, for the first time, the ability of a microbial eumelanin, biotechnologically produced and purified by *Streptomyces nashvillensis* DSM 40314, to act as a protective agent against oxidative stress, UV radiation damage, and cytotoxicity associated with advanced glycation end products (AGEs) in a human keratinocyte cell model. Also, the effect of amyloid fibrils on the structural and biological properties of microbial melanin has been investigated for the first time, and the results indicated that the addition of an amyloid scaffold promotes an increase of melanin antioxidant activity and provides greater cellular protection against UV irradiation and AGE-induced cytotoxicity, key events associated with skin damage and aging. This study provides new insights into the functional role of amyloid in enhancing the protective properties of biotechnologically produced microbial melanin, highlighting its potential as a substitute for animal-derived eumelanin.

## Graphical abstract

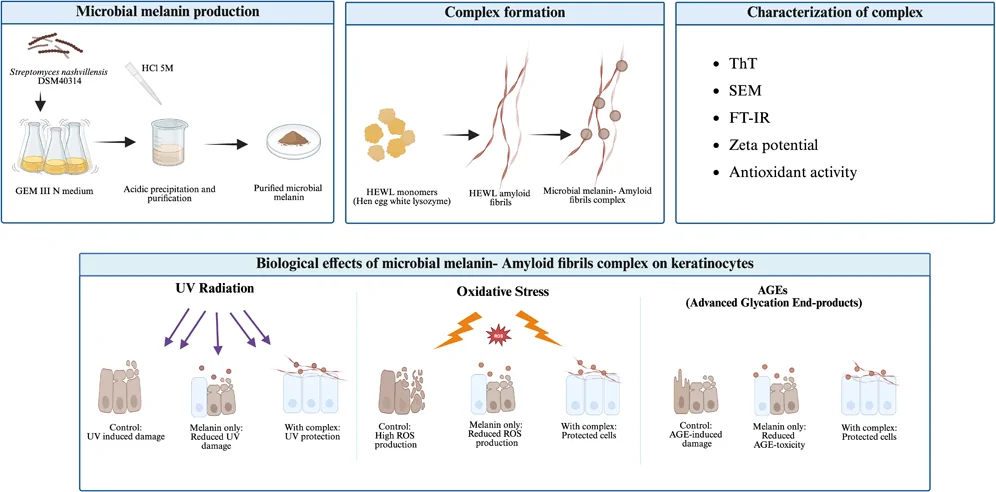

## 1 Introduction

Melanins are polymeric pigments found in the animal, plant, and microbial kingdoms. Depending on their structure, they may appear dark brown (eumelanin, pyomelanin, and allomelanin), yellow, or red (pheomelanin). They possess distinctive physicochemical properties, including high thermal stability, strong UV–visible light absorption, antioxidant and redox activities, and important biological properties such as high biocompatibility, biostability, antimicrobial activity, and lack of antigenicity.^1–3^ Consequently, melanins are currently employed as antioxidants and antitumor agents, as radioprotective molecules. Furthermore, these polymers play a pivotal role in fabricating 3D-bioprinted biocompatible scaffolds for tissue engineering as well as in synthesizing nanoparticles designed for antiviral and antimicrobial drug delivery.^1^ Despite their huge potential, the real applications of melanins are restricted by the fact that they are mainly produced by difficult extraction and purification procedures from animal tissues, such as cephalopod ink sacs (e.g., *Sepia officinalis*), or by chemical synthesis that lead to pigments whose structures are not natural-like. Besides, these processes are expensive, not sustainable, slow, and not environmentally friendly. Because mammalian-like melanin (eumelanin and pheomelanin) should be preferred for biological and biomedical studies, new strategies to produce natural-like pigments possessing animal-like structures are needed. In this respect, the biotechnological production of microbial melanin has gained increasing attention as a sustainable, reliable, reproducible, and environmentally friendly alternative. In particular, some *Streptomyces* strains produce melanin, whose structure resembles that of mammalian eumelanin, that is released into the surrounding medium as an extracellular product.^3–5^ Recently, the biotechnological production of microbial melanin by *Streptomyces nashvillensis* DSM 40314 has been enhanced by optimizing the pH and temperature conditions for bacterial growth and by adding to the growth medium metal ions as cofactors or lignocellulose substrates that provide phenolic precursors.^3,6–9^ An optimized downstream process allowed the achievement of a highly purified pigment, which was extensively characterized by UV–visible, FTIR, and NMR analyses, showing an eumelanin-like structure. The pigment also showed high thermal stability and good UV–visible light shielding capacity, as well as redox, antimicrobial, and antioxidant properties.^3,7–9^ However, this microbial melanin has not yet been tested in cell systems to evaluate its biological effects as an alternative to animal-derived pigments. Moreover, the role of amyloid fibrils in modulating the microbial melanin biological activities has not been explored either. Amyloid fibrils are generated through the self-assembly of proteins into highly ordered, macromolecular aggregates characterized by a distinct fibrillar morphology and shared structural features. Although amyloid structures have largely been described as detrimental and pathological in many contexts, they are now widely accepted to be functional molecular scaffolds in nature. Indeed, their supramolecular structure confers useful properties, including superior material characteristics (e.g., physicochemical stability and high rigidity/elasticity) and/or chemical functions (e.g., catalytic scaffolding, gene regulation, hormone storage, and molecular recognition).^10–13^ Amyloid fibrils also seem to be involved in melanin synthesis. In mammals, melanin is produced through a process called melanogenesis, which involves oxidation of the amino acid L-tyrosine followed by polymerization. During this process, functional amyloid fibrils, in the form of an amyloid matrix of melanosomal proteins (Pmel17), have been found to act as a physical scaffold to sequester potentially harmful intermediate molecules of pigment synthesis and to provide mechanical support for the biosynthetic process.^14–16^ In this context, the melanosomal glycoprotein Pmel17 constitutes an indispensable component of melanogenesis. Following sequential proteolytic cleavages, the luminal domain of Pmel17 spontaneously self-assembles into functional amyloid fibrils, which subsequently serve as a structural scaffold for melanin deposition. Although Pmel17 fibrils have been proposed to physically keep potentially harmful intermediates of melanogenesis away from the cytosol, their role in the structure and properties of melanin is still poorly understood.^17–20^ Recent findings have shown that Pmel17 amyloid fibrils affect catalytic activities of catecholamines during oxidative coupling (an in vitro alternative of melanogenesis), along with the modulation of water dispersibility and morphology of the final melanin product.^21,22^ Although natural melanins are typically distinguished from chemically synthesized ones, the addition of an amyloid scaffold to a non-animal-like synthetic pigment has been recently shown to increase the pigment spin density, thus increasing water dispersibility, bandgaps, and radical-scavenging properties.^23^

While previous literature on microbial melanin by *Streptomyces* strains has focused mainly on production optimization and physicochemical characterization,^3,7–9^ its functional biocompatibility and biological effects in human cell systems have remained completely unexplored. To bridge this gap, the aim of this study was to evaluate the functional and biological effects of microbial melanin, biotechnologically produced and purified by *Streptomyces nashvillensis* DSM 40314, whose structure resembles that of animal eumelanin, in a human cell system. This study provides, for the first time, a functional characterization of microbial eumelanin produced by a *Streptomyces* strain on human keratinocytes, demonstrating its strong antioxidant and photoprotective effects. Furthermore, setting this study apart from existing literature on functional amyloids, the effect of amyloid fibrils on the structural and biological properties of microbial melanin has been investigated for the first time by using a comprehensive combination of biophysical and cell biology techniques. Specifically, we have evaluated the ability of microbial melanin and the melanin-amyloid complex to protect human keratinocytes from oxidative stress, UV-induced damage, and cell toxicity associated with advanced glycation end products (AGEs), which are key markers of skin damage and aging. This study provides a first insight into the functional role of amyloid in enhancing the protective biological properties of microbial melanin in mammalian systems and identifies a beneficial role for microbial melanin, highlighting its potential as a substitute for animal-derived eumelanin.

## 2 Materials and Methods

### 2.1 Materials

Hen egg white lysozyme, human insulin, d-ribose, Thioflavin T (ThT), 1,1-diphenyl-2-picrylhydrazyl (DPPH), 2′,7′-dichlorofluorescin diacetate (DCFH-DA), Trypan Blue, glucose, and synthetic melanin standard were purchased by Sigma-Aldrich (USA). Complex nutrients like yeast and malt extracts were obtained from Himedia Laboratories (India). All other chemicals were of analytical grade.

### 2.2 Melanin Production and Purification

The microbial melanin pigment was produced by *Streptomyces nashvillensis* DSM 40314 (DSMZ, Germany) in a 1-L shake flask on GEM III N medium (glucose, 12.0 g/L; yeast extract, 6.0 g/L; malt extract, 30.0 g/L; NaH_2_PO_4_·H_2_O, 5.8 g/L; Na_2_HPO_4_, 8.2 g/L; pH 7.0), for 96 h as previously reported.^6,7^ The pigment was precipitated from the clarified broth supernatant by adding 5.0 M HCl up to a pH of 1.5 and then washed several times with 5.0 M HCl and Milli-Q water, as previously reported.^7–9^ Once dried, the pureness of the pigment was determined by measuring the absorbance between 200 and 500 nm (Spectrophotometer V-530, Jasco, Japan) after dissolving the sample in 0.1 M NaOH, which was also used as a blank, and then calculating the pureness on the base of a synthetic melanin standard calibration curve, as previously reported.^7–9^

### 2.3 Cell Cultures and Treatments

Human keratinocyte cell line HaCaT (ATCC CRL-2309) was cultured in Dulbecco's Modified Eagle Medium (DMEM) (11965092, Gibco) supplemented with 10% FBS (A5670701, Gibco), 2.0 mM glutamine (X0550, Microgem), 100 units/mL penicillin, and 100 mg/mL streptomycin (A001, Himedia) in a 5.0% CO_2_ humidified environment at 37 °C. Cells were grown for 18 h before the treatments. For evaluation of UV-induced damage, HaCaT cells were pretreated for 5 h with 0, 25, 50, and 100 μg/mL microbial melanin, with and without amyloid fibrils in a 1:1 ratio and then exposed to a UV source (40 mJ/cm^2^) for 10 min.

### 2.4 Trypan Blue Assay

Trypan Blue assay was performed to evaluate the number of dead cells in the different experimental groups. Trypan Blue is strictly excluded by viable cell membranes; consequently, its intracellular presence serves as a definitive marker of membrane disruption. Once inside the cytoplasm, the dye imparts a distinct dark blue coloration to the compromised cells. Briefly, cells were seeded in a 6-well plate at a density of 2 × 10^5^ cells/well in medium supplemented with 10% FBS and incubated for 18 h at 37 °C prior to treatment. Post-treatment, cells were harvested and mixed with an equal volume of 0.4% (v/v) Trypan Blue (10 μL each). Both viable (unstained) and dead (blue-stained) cells were subsequently quantified. The results were expressed as average values with standard deviations from three independent experiments carried out in triplicate.

### 2.5 Preparation and Quantification of HEWL Amyloid Fibrils

Hen egg white lysozyme (HEWL) was dissolved in a solution of potassium phosphate buffer (20 mM, pH 6.3) containing 2.0 M GdnHCl up to a concentration of 2.0 mg/mL. The concentration of HEWL was estimated by absorbance at 280 nm using an extinction coefficient of 38,904 M^–1^ cm^–1^. The HEWL amyloid fibrils were produced in vitro according to an aggregation process well described in the literature by Kang and co-workers.^23^ The solution of HEWL was incubated at 60 °C under continuous stirring conditions for 4 h; then, it was centrifuged for 10 min at 10,000 × *g* to remove the remaining salts and the monomeric HEWL from the solution. The HEWL aggregate was washed five times with ultrapure Milli-Q water by centrifugation, and the concentration of monomeric HEWL in each discarded supernatant was measured to evaluate the final concentration of HEWL fibrils. Aliquots of protein were collected in sterile conditions and immediately analyzed to verify amyloid fibril formation by ThT fluorescence assay.

### 2.6 ThT Fluorescence Assay

Thioflavin T is a small molecule that produces strong and specific fluorescence upon binding to amyloid fibrils. To assess amyloid fibril formation, ThT fluorescence (λ_ex_ 450 nm/λ_em_ 482 nm) was monitored after addition of the fluorophore to the HEWL samples. Working concentrations were 10 μM for HEWL and 30 μM for ThT. Fluorescence measurements were performed by using a spectrofluorometer (PerkinElmer Life Sciences LS 55, PerkinElmer, Inc., Waltham, MA, USA).

### 2.7 Scanning Electron Microscopy Measurements

HEWL amyloid fibrils incubated for 48 h in the absence and presence of microbial melanin (at 1:1 and 1:2 ratios) were analyzed in duplicate by scanning electron microscopy (SEM). Each sample (200 μL) (0.25 mg/mL) was first placed onto a cover glass, dried at 60 °C overnight, and then placed on a carbon adhesive film support. The samples were metallized by a sputter coater (Denton Vacuum DeskV) with a thin layer of Au–Pd and then observed with a field emission scanning electron microscope (Nova NanoSEM 450; FEI Instruments) operating at an accelerating voltage of 3.0 kV, with an Everhart–Thornley detector and through-the-lens detector for higher magnifications.

### 2.8 Fourier-Transform Infrared Spectroscopy Measurements

FT-IR spectra of the microbial melanin in the presence and absence of HEWL amyloid fibrils were recorded between 4000 and 400 cm^–1^ (FTIR-4700 instrument, Jasco, Japan) by using 600 scans and a resolution of 2.0 cm^–1^, at room temperature, after grinding 1.0 mg of each freeze-dried sample with KBr powder (199.0 mg) in an agate mortar and then pressing them to obtain discs, with a procedure similarly to what previously reported.^8,9^

### 2.9 Zeta Potential Measurements, Particle Size, and Polydispersity

Zeta potential measurements of the microbial melanin in the absence and presence of HEWL amyloid fibrils in 1:1 and 1:2 ratios were carried out at 25 °C in triplicate by a Zetasizer instrument (Zetasizer Nano-ZSP, Malvern, Worcestershire, UK) by calculating the electrophoretic mobility by using the Henry equation (Zetasizer software v7.12). For all measurements, samples were prepared in potassium phosphate buffer (20.0 mM, pH 6.6) at a 1.0 mg/mL melanin concentration. The particle size and polydispersity index (PDI) were measured by dynamic light scattering using the same instrument.

### 2.10 Antioxidant Activity

The antioxidant activity of the microbial melanin in the absence and presence of HEWL amyloid fibrils in 1:1 and 1:2 ratios was measured by DPPH (1,1-diphenyl-2-picrylhydrazyl) assay.^24^ For all measurements, samples were prepared at a 5.0 mg/mL melanin concentration in potassium phosphate buffer (20.0 mM, pH 6.6). For the assays, 10 μL of the sample was mixed with 100 μL of a 67.5 μM solution of DPPH in methanol and 190 μL of methanol in a 96-well microplate, in triplicate. The reaction mixtures were incubated for 1 h at room temperature in the dark. Subsequently, the absorbance at 517 nm was recorded using a UV–visible spectrophotometer equipped with a microplate reader (Varian Cary 50 UV–visible spectrophotometer, USA). For the control sample, 10 μL of phosphate buffer (20.0 mM, pH 6.6) was used in replacement of the sample, while for the blank only methanol (290 μL) and 10 μL of phosphate buffer were mixed. The inhibition percentage of radical scavenging activity was calculated by using the following equation:

1 I(%)=[(Ac−As)/Ac]×100

where Ac is the absorbance of the control and As is the absorbance of the sample.

### 2.11 Lactate Dehydrogenase Activity Assay

The activity of lactate dehydrogenase (LDH) was evaluated in the cell medium using an Abbott Alinity c Lab Chemistry Analyzer. Cells were seeded in FBS-free medium in a 24-well plate at a density of 4.0 × 10^4^ cells/well for 18 h at 37 °C before treatment. Relative enzyme activity was determined in accordance with the manufacturer’s instructions.

### 2.12 Detection of Intracellular ROS

Intracellular ROS were detected by the oxidation-sensitive fluorescent probe 2′,7′-dichlorofluorescin diacetate (DCFH-DA) as previously reported.^25^ Cells were grown in 24-well plates (4.0 × 10^4^ cells/well), preincubated with melanin samples for 5 h and exposed to 600 μM H_2_O_2_ for 6 h or 30 μM AGEs for 24 h. As the control, untreated cells and cells exposed to H_2_O_2_ alone were used. After incubation with the samples, 20 μM DCFH-DA was added to the wells for 30 min, and then the cells were washed twice with PBS buffer and lysed with 0.5 M Tris-HCl 1.0% SDS buffer, at pH 7.6. The nonfluorescent probe DCFH-DA was oxidized to the highly fluorescent molecule 2′,7′-dichlorofluorescein (DCF). The resulting DCF fluorescence intensity was quantified using a PerkinElmer Life Sciences LS 55 spectrofluorometer, with excitation and emission wavelengths set at 488 and 530 nm, respectively. Data were then expressed as average values with standard deviations from five independent experiments carried out in triplicate.

### 2.13 AGE Formation

AGE formation was performed by in vitro glycation of 1.0 mg/mL human insulin with 0.5 M d-ribose as the glycating agent. Glycation reaction was performed in 50 mM NaH_2_PO_4_ buffer, at pH 7.0, filtered through a 0.22 μm membrane and incubated at 37 °C in sterile conditions. The AGE formation was monitored by intrinsic fluorescence (λ_ex_ 320 nm/λ_em_ 410 nm) over time.^26,27^ The protein was fully glycated after 7 days of incubation at 37 °C, and this sample was used for cellular assays.

### 2.14 Statistical Analysis

Statistical analyses were performed using Stata software (Version 13.0; StataCorp LP, College Station, TX, USA). When the analysis of variance (ANOVA) indicated significant treatment effects, a Tukey’s post hoc test was applied for pairwise comparisons. All data are represented as the mean values with standard deviations. Statistical significance was set at *p* < 0.05.

## 3 Results and Discussion

### 3.1 Cell Toxicity Evaluation of Microbial Melanin in Human Keratinocytes

The microbial melanin was biotechnologically produced in shake flasks on GEM III N medium by *Streptomyces nashvillensis* DSM 40314, as previously reported.^7^ The final concentration of the pigment was similar to previous published values (0.73 ± 0.01 g/L), and the pureness, determined after the downstream procedure, was always higher than 85.0 ± 0.1%. As mammalian melanin is known to be nontoxic for cells even at high concentrations, first the cell toxicity of the microbial melanin was studied in a human epidermal keratinocyte cell line (HaCaT cells) to optimize the experimental conditions. Following this aim, HaCaT cells were exposed to different concentrations of microbial melanin (0, 25, 50, and 100 μg/mL) for 24 and 48 h, and the cell toxicity was evaluated by estimation of the dead/living cell ratio by the Trypan Blue assay and lactate dehydrogenase (LDH) assay. No difference in the number of living and dead cells was observed in cells exposed to the different melanin concentrations after both 24 and 48 h of incubation, thus suggesting that melanin did not induce cell toxicity in our experimental conditions (Figure 1A). The same results were obtained by measuring the activity of LDH in the cell medium, which is a primary marker of cytotoxicity. Indeed, no activity of LDH was detected for all experimental groups, indicating the absence of cell damage (data not shown). To assess changes in cell morphology after melanin exposure, the experimental groups were also examined using phase contrast microscopy (Figure 1B). In accordance with the Trypan Blue assay, the analyses showed that, independent of the melanin concentration used (up to 100 μg/mL) and exposure time, cells exposed to microbial melanin did not display any qualitative or quantitative alterations. Moreover, following melanin exposure, the cell medium became clarified after a few minutes, and small black granules were visible inside the cells. These data are similar to those reported by Ha and co-workers^23^ for synthetic melanin, suggesting that microbial melanin by *Streptomyces nashvillensis* DSM 40314 might be easily internalized in mammalian cells without affecting cell viability, even at high concentrations.

**Figure 1:**
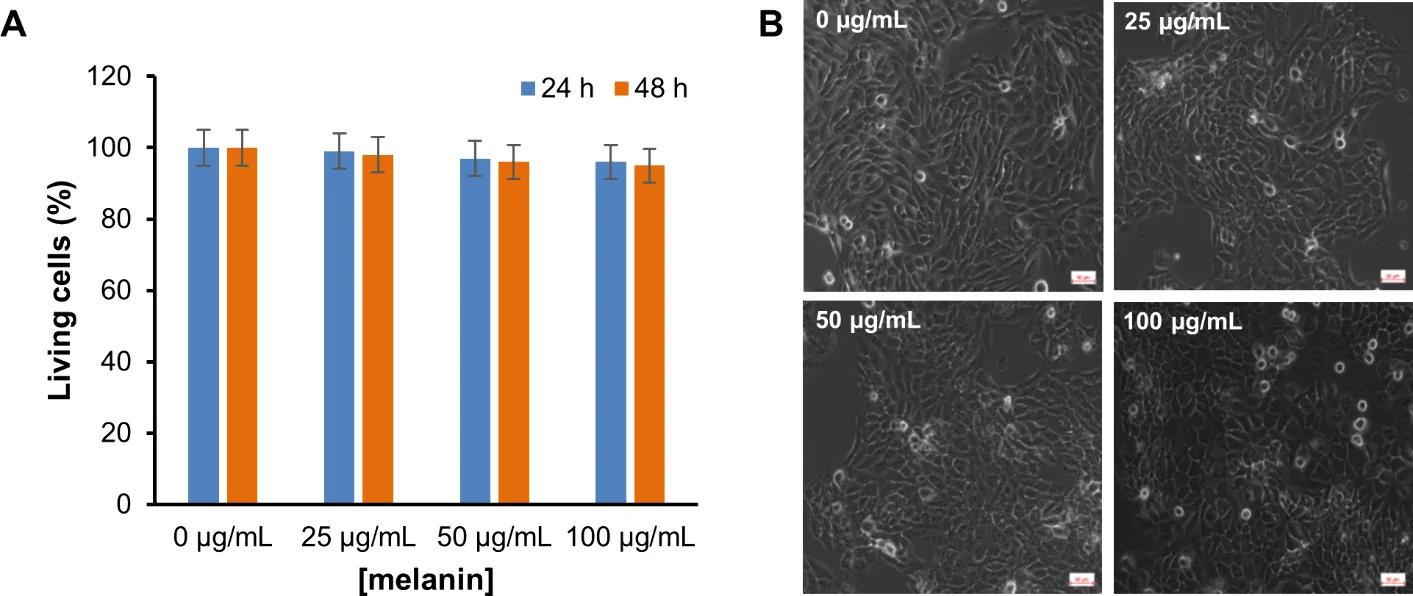
Evaluation of microbial melanin cytotoxicity in human keratinocytes. (A) Evaluation of living cells after 24 and 48 h of incubation and (B) phase contrast microscopy images at 48 h of incubation for different concentrations of microbial melanin in HaCaT cells. Scale bar: 50 μm. Data are expressed as average number ± SD from triplicate wells from three separate experiments. Other experimental details are described in the Materials and Methods section.

### 3.2 Microbial Melanin-Amyloid Fibril Complex Formation and Characterization

In order to evaluate the ability of microbial melanin to bind amyloid fibrils and to study the properties of the melanin-amyloid complex, hen egg white lysozyme (HEWL) has been used as a protein model to form the amyloid scaffold. The HEWL amyloid fibril formation has been well characterized in vitro, and for this reason, it is a widely used protein model for studies involving amyloid fibrils.^28–30^ Specifically, as amyloid fibrils share a common ultrastructure independently from the protein involved, HEWL amyloid fibrils have been used in similar studies to mimic amyloid Pmel17 in melanin formation.^21^ Indeed, Pmel17 and HEWL fibrils have been shown to exhibit behavior comparable to that of amyloid scaffolds in melanin-like pigment synthesis.^30^

First, the ability of HEWL fibrils to further aggregate in the presence and absence of melanin was monitored by ThT fluorescence assay, following the amyloid formation for 7 days (Figure 2A). ThT is a very sensitive and specific dye, and its fluorescence is directly associated with the presence of amyloid fibrils in solution. In this experiment, 25 μg/mL melanin was incubated in the absence (M) and presence of 25 (M + A1) and 50 μg/mL (M + A2) amyloid fibrils (1:1 and 1:2 melanin-amyloid ratios). The HEWL samples at 25 (A1) and 50 μg/mL (A2) in the absence of melanin have been also analyzed. As expected, HEWL samples incubated in the absence of melanin (A1, A2) showed an increase in ThT fluorescence over time, thus suggesting that the samples further aggregate, forming larger amyloid fibrils in solutions. Interestingly, in the HEWL samples coincubated with melanin (M + A1, M + A2) no increase in fluorescence over time was recorded at both 1:1 and 1:2 melanin-amyloid ratios (Figure 2A). These data indicated that the presence of melanin inhibited the further aggregation of HEWL amyloid fibrils, suggesting a direct interaction between microbial melanin and amyloid fibrils. The same experimental groups were also analyzed by scanning electron microscopy (SEM), which showed that microbial melanin was embedded within the HEWL amyloid fibrils, further suggesting the formation of a melanin-amyloid complex (Figure S1). To test the latter hypothesis, we also performed FTIR analyses on melanin in the presence and absence of HEWL amyloid fibrils (Figure 2B). FT-IR analyses showed possible interactions between the pigment and the amyloid structure, with signals that resulted in a merge of the two molecules and whose intensities clearly changed (Figure 2B). Specifically, the strong broad peak of the microbial melanin in the 3500–3200 cm^–1^ range indicating the stretching of −OH and −NH groups,^32^ perfectly merged with the peaks of the HEWL amyloid fibrils in the same region, giving rise to averaged, merged signals of lower intensity in the complex. Similarly, the signals due to the C=O stretching in the amide I region (1700–1600 cm^–1^), present in both microbial melanin and HEWL fibril samples, reduced in intensity in the melanin-amyloid fibril complex at both ratios. Differently, the intensity of the signals in the amide II region (1600–1500 cm^–1^), which are due to C–N and N–H stretching, was slightly increased (Figure 2B). These data suggest that the interaction between microbial melanin and amyloid fibrils could take place on their respective polar groups.

**Figure 2:**
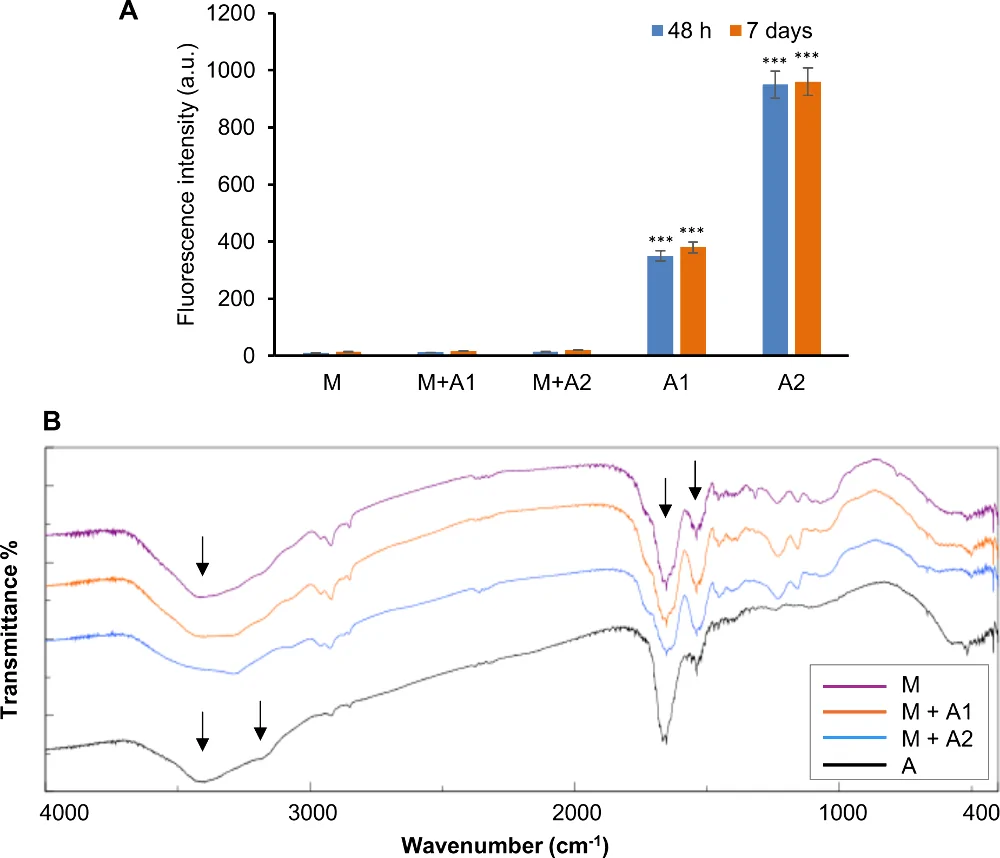
Microbial melanin-amyloid complex formation. (A) ThT fluorescence intensity of HEWL fibrils incubated with 25 μg/mL microbial melanin at two different concentration melanin:amyloid ratios of 1:1 (M + A1) and 1:2 (M + A2). A1 and A2 represent amyloid fibrils incubated in the absence of melanin; M represents microbial melanin incubated in the absence of amyloid fibrils. The ThT fluorescence was recorded at 48 h and 7 days of incubation at room temperature. ***p < 0.05 versus the sample in the presence of melanin. (B) FTIR spectra of microbial melanin in the absence (M) and presence of HEWL amyloid fibrils at the two different ratios (M + A1 and M + A2). A: amyloid fibrils incubated in the absence of melanin. The main signals are indicated by arrows.

Moreover, the FTIR spectra of the melanin species, recorded both in the presence and absence of amyloid fibrils, closely resembled the sum of the individual spectra of melanin and the corresponding amyloid species. These data suggest that melanin binding to the amyloid scaffold stabilizes the fibril surfaces and inhibits further aggregation without causing remodeling or disassembly, as corroborated by the ThT analysis. Similar results have been reported regarding the interactions between HEWL fibers and dopamine during the investigation of polydopamine synthesis and polydopamine-fiber complex formation.^30^ To further investigate the interaction between microbial melanin and amyloid fibrils, dynamic light scattering (DLS) and zeta potential analyses were performed on melanin in the absence and presence of HEWL amyloid fibrils (1:1 and 1:2 ratios) in a potassium phosphate buffer, pH 6.6 (Table 1).

**Table 1:** Zeta Potential Evaluation and Particle Size Distribution of the Microbial Melanin in the Absence and Presence of HEWL Amyloid Fibrils (1:1 and 1:2 Ratios)

	Particle Size (nm)	Zeta Potential (mV)	PDI
Melanin	505.0 ± 11.5	–33.3 ± 11.7	0.513 ± 0.110
Melanin-amyloid 1:1	1074.0 ± 21.5	–34.7 ± 5.9	0.859 ± 0.120
Melanin-amyloid 1:2	2240.0 ± 17.5	–36.2 ± 9.4	0.715 ± 0.100
HEWL amyloid	1080.0 ± 20.1	–26.8 ± 10.8	0.788 ± 0.200

Melanin particles exhibited an average hydrodynamic diameter of 505.0 ± 11.5 nm and a zeta potential of −33.3 ± 11.7 mV, values that are consistent with moderately stable colloidal aggregates. HEWL amyloid fibrils displayed larger assemblies (1080.0 ± 20.1 nm) and a less negative zeta potential value (−26.8 ± 10.8) under the same conditions. In the microbial melanin-amyloid complex, the hydrodynamic diameter increased significantly, reaching values of up to 2240.0 ± 17.5 nm in the case of the 1:2 ratio, and the zeta potential remained consistently negative (−34.7 ± 5.9 to −36.2 ± 9.4 mV) regardless of the ratio, indicating the formation of a stable melanin-amyloid complex. Polydispersity index (PDI) analyses of the melanin samples in the presence of amyloid fibrils at the 1:1 ratio showed a higher PDI value (0.859 ± 0.120) than either the melanin or the fibrils alone, indicating the greater size heterogeneity of the melanin-amyloid assemblies. Also, the slightly lower PDI value in the melanin-amyloid complex at the 1:2 ratio (0.715 ± 0.100) suggested that the increase of the fibril amount in the sample led to a system that was more dominated by the HEWL aggregation state than by the microbial melanin one. It is difficult to rationalize how the higher PDI values in the melanin-amyloid complex may affect cellular uptake or biological performance. However, we can hypothesize that, irrespective of particle size, the presence of the amyloid scaffold may modulate the structural properties of melanin particles, thereby altering their biological function. This effect appears to be independent of particle size, in line with analogous data reported for synthetic melanin complexed with amyloid scaffolds, which exhibits enhanced internalization in HaCaT cells.^23^ Overall, these findings demonstrate a ratio-dependent co-interaction between melanin and amyloid fibrils that might affect the chemical-physical properties of melanin, similarly to what was previously demonstrated for the synthetic melanin and amyloid fibril complex.^23^

### 3.3 Antioxidant Properties of Microbial Melanin and Melanin-Amyloid Complex

Melanin is a powerful antioxidant due to its stable intrinsic unpaired electrons, which readily scavenge reactive species and free radicals, and it is known to protect biological systems against reactive oxygen species (ROS)-mediated damage.^32,33^ In this respect, the ability of amyloid fibrils to interfere with microbial melanin antioxidant properties was evaluated by measuring the scavenging activity on the free radical 1,1-diphenyl-2-picrylhydrazyl (DPPH). The DPPH assay was performed on melanin in the absence and presence of the amyloid fibrils at two different ratios (1:1 and 1:2) (Figure 3).

**Figure 3:**
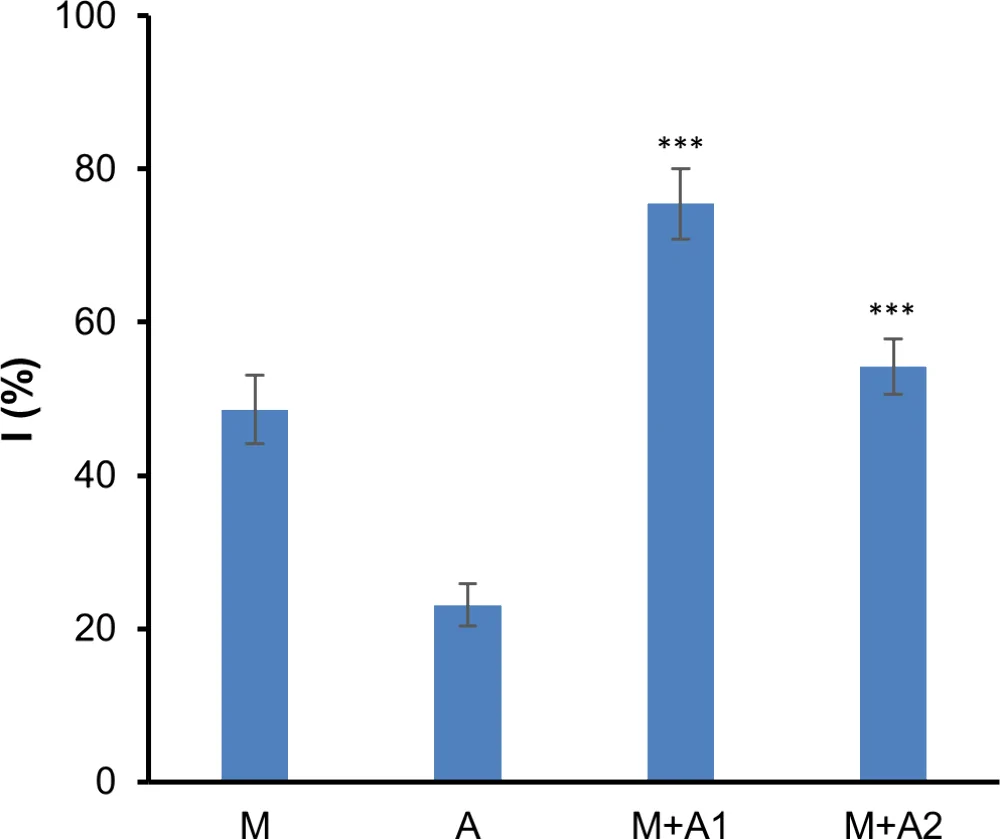
Antioxidant activity. The scavenging ability was evaluated by DPPH assay for microbial melanin in the absence (M) and presence of HEWL amyloid fibrils at two different concentration melanin:amyloid ratios of 1:1 (M + A1) and 1:2 (M + A2). Data are expressed as the average number ± SD from triplicate wells from three separate experiments. Other experimental details are described in the Materials and Methods section. ***p < 0.05 versus melanin.

The results showed that the microbial melanin exhibited moderate DPPH radical scavenging activity (48.6 ± 4.5%), while amyloid fibrils alone showed very limited activity (23.1 ± 2.8%). Interestingly, the melanin-amyloid fibril complex at the 1:1 ratio showed a 55.3% enhancement in antioxidant activity (75.5 ± 4.6%), a value that clearly depends on the additive antioxidant actions of the pigment and the fibrils. Considering that the theoretical value would be about 71.7% (sum of 48.6% and 23.1%), the higher antioxidant activity observed for the complex is consistent with the hypothesis that the presence of amyloid fibrils enhances the antioxidant properties of microbial melanin. At a higher amyloid concentration (1:2 ratio), the scavenging activity was reduced to 54.2 ± 3.6%; the decreased value is likely due to the higher amyloid content in the sample. Similar data were also obtained for synthetic melanin in the presence of amyloid fibrils,^23^ thus suggesting that the presence of amyloid fibrils can contribute to improve the antioxidant ability of the pigment.

To further test the ability of the amyloid fibrils to affect the antioxidant properties of the microbial melanin, the antioxidant effect in human keratinocytes was also explored. At first, the toxicity of different concentrations of microbial melanin and the melanin-amyloid complex was evaluated by Trypan Blue assay, lactate dehydrogenase (LDH) assay, and phase contrast microscopy to set up the experimental conditions. HaCaT cells were exposed to different concentrations (0, 25, 50, and 100 μg/mL) of melanin and the melanin-amyloid complex (1:1 ratio) for 48 h (Figure 4). No difference in the number of living and dead cells was observed in cells exposed to the melanin-amyloid complex, indicating that the presence of amyloid fibrils in the complex does not induce cell toxicity (Figure 4A). The same result was obtained by measuring the activity of LDH in the cell medium, which is a primary marker of cytotoxicity. Indeed, no activity of LDH was detected for all experimental groups, indicating absence of cell damage (Figure 4B). These data were also confirmed by phase contrast microscopy analyses, in which cells exposed to the microbial melanin-amyloid complex did not display any qualitative or quantitative alterations up to a 100 μg/mL concentration (Figure 4C).

**Figure 4:**
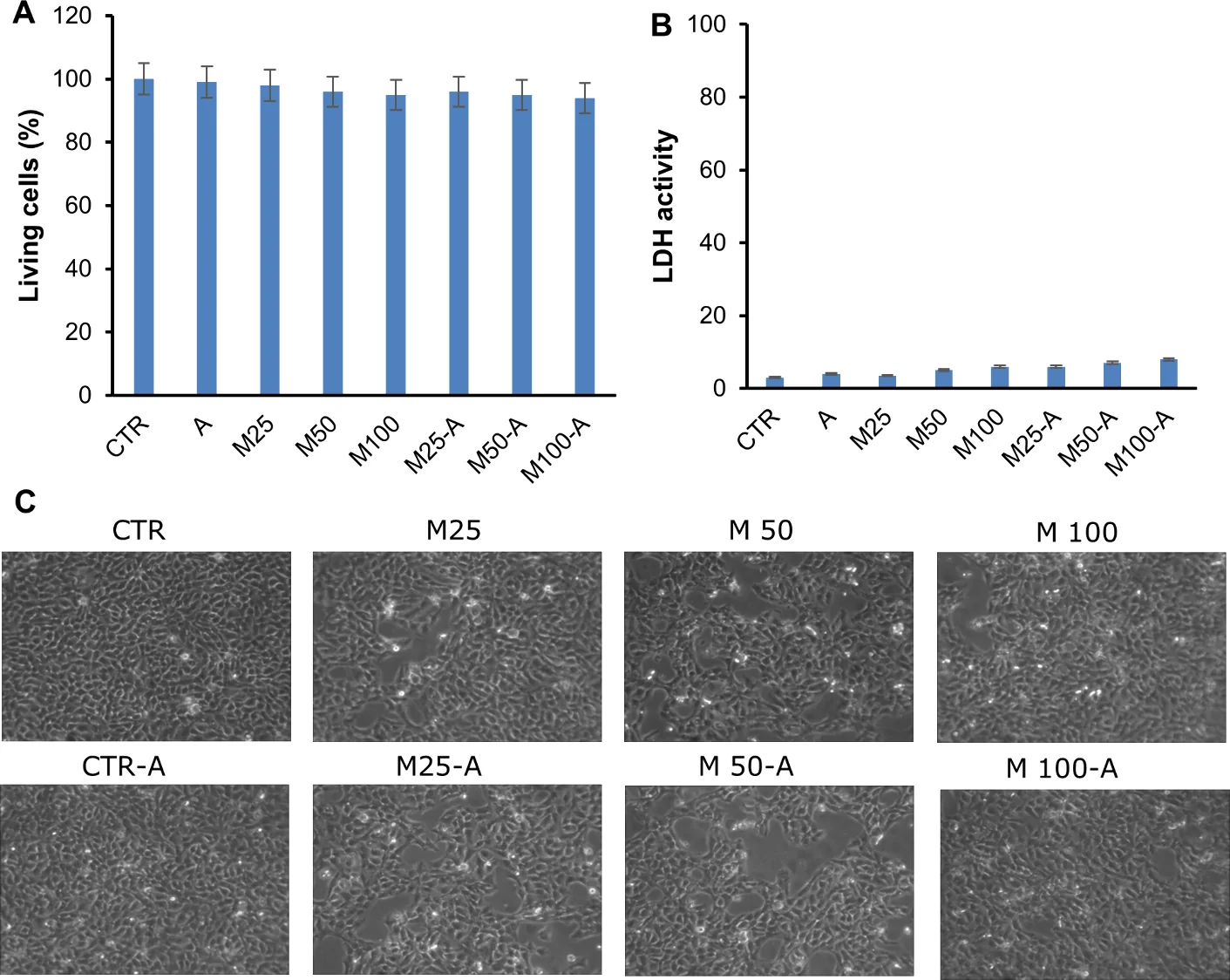
Evaluation of microbial melanin-amyloid complex cytotoxicity in human keratinocytes. (A) Evaluation of living cells, (B) LDH activity, and (C) phase contrast microscopy images for HaCaT exposed to 25, 50, and 100 μg/mL microbial melanin (M25, M50, and M100) and melanin-amyloid complex at a 1:1 ratio (M25-A, M50-A, and M100-A) for 48 h. CTR: untreated cells; CTR-A: cells exposed to amyloid fibrils alone. Scale bar: 50 μm. Data are expressed as the average number ± SD from triplicate wells from three separate experiments. Other experimental details are described in the Materials and Methods section.

Moreover, also in this case, we observed that following exposure to the melanin-amyloid complex, the cell culture medium cleared within a few minutes and small black granules became visible inside the HaCaT cells, indicating that the complex was efficiently internalized.

Given the absence of toxicity observed for the melanin-amyloid complex at all the tested concentrations and considering the antioxidant properties of microbial melanin, the ability of the melanin-amyloid complex, in comparison with microbial melanin, to protect HaCaT cells by oxidative stress induced by H_2_O_2_ was explored. In this experiment, cellular damage in HaCaT cells preincubated with melanin samples for 5 h was evaluated after exposure to 600 μM H_2_O_2_ for 6 h (Figure 5). The cell toxicity was assessed in these experimental groups by the lactate dehydrogenase (LDH) assay performed in the cell medium. Indeed, LDH release into the cell medium is a primary marker of cytotoxicity, indicating cell membrane damage caused by oxidative stress-induced lipid peroxidation.^34,35^ Oxidative stress disrupts cell integrity, causing this intracellular enzyme to leak into the culture medium, where its activity is measured to quantify cell death. As expected, the treatment with H_2_O_2_ promoted a marked increase in LDH release after 6 h of incubation, suggesting strong membrane damage. Differently, samples preincubated with different melanin samples showed strongly reduced LDH levels (65.0–80.0% reduction), suggesting that the microbial melanin was able to counteract H_2_O_2_-induced cell damage in human keratinocytes. Notably, the protective effect was even stronger in the presence of the melanin-amyloid complex, which showed more than 90% cellular protection (Figure 5A). Since an increase in LDH in the medium often correlates with elevated ROS production, oxidative stress was also assessed through the DCFH fluorescence assay (Figure 5B). H_2_O_2_ treatment promoted a clear increase in DCF fluorescence after 6 h of incubation, indicative of ROS production. By contrast, cells preincubated with microbial melanin showed strongly reduced ROS levels (70.0–80.0% reduction) in a concentration-dependent manner, suggesting that the pigment is able to counteract the H_2_O_2_-induced ROS production in human keratinocytes. In addition, the melanin-amyloid complex displayed a stronger protective effect, inhibiting ROS production by up to 99.0%.

**Figure 5:**
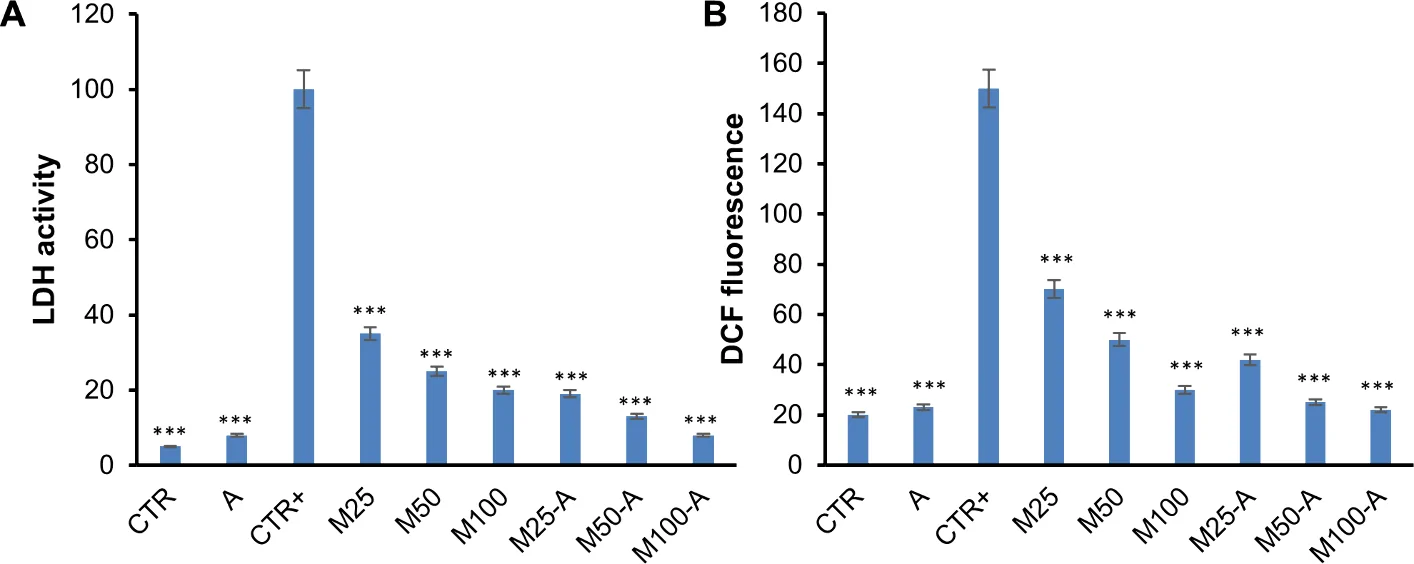
Protective effect of microbial melanin against oxidative stress induced by H_2_O_2_. HaCaT cells were pretreated with 25, 50, and 100 μg/mL microbial melanin (M25, M50, and M100) and melanin-amyloid complex at a 1:1 ratio (M25-A, M50-A, and M100-A) for 5 h and then exposed to 600 μM H_2_O_2_ for 6 h. (A) LDH assay and (B) DCFH-DA assay for all experimental groups. CTR: untreated cells; CTR-A: cells exposed to amyloid fibrils alone; CTR+: cells exposed to 600 μM H_2_O_2_. Data are expressed as the average intensity ± SD from triplicate wells from five separate experiments. Other experimental details are described in the Materials and Methods section. ***p < 0.05 versus CTR+.

These results are in agreement with the DPPH analysis, which identified higher antioxidant activity for the melanin-amyloid complex. It should be emphasized that, in the presence of H_2_O_2_ and the melanin-amyloid complex, LDH and ROS levels were similar to those of untreated cells. These data suggest that, while H_2_O_2_ promotes a strong LDH release and ROS production after 6 h, clear reduction was observed in cells preincubated with melanin and the melanin-amyloid complex, indicating that they effectively counteracted H_2_O_2_-induced oxidative stress and help to maintain cellular redox homeostasis. Interestingly, the presence of amyloid fibrils contributed to enhance the antioxidant ability of microbial melanin. This effect could be explained by the observation that melanin, when integrated into amyloid fibrils, exhibits an increased intrinsic spin density, which may be responsible for its enhanced radical-scavenging capacity against ROS.^23^

### 3.4 Effect of Melanin-Amyloid Complex as a Cytoprotective Agent against UV Damage

UV radiation is harmful, and excessive exposure to it can lead to various skin cancers and other adverse health effects. Radiation damage is one of the most common causes of harmful changes in the human body, and skin pigmentation is considered the main photo-protective factor against UV and ionizing radiation.^36,37^ Indeed, light absorption is one of the most prominent features of melanin polymers, due to their highly conjugated structures that are able to protect against light radiation in humans.^38–40^ To test whether amyloid fibrils would also facilitate the melanin cytoprotective capabilities from UV irradiation, human keratinocytes were pretreated with microbial melanin and the melanin-amyloid complex and exposed to UV irradiation. In particular, HaCaT cells were pretreated for 5 h with 0, 25, 50, and 100 μg/mL microbial melanin, with and without amyloid fibrils in a 1:1 ratio, and then exposed to UV light for 10 min. To assess the protective role of melanin and the melanin-amyloid complex, after UV exposure the cells were analyzed by Trypan Blue assay to estimate the number of living and damaged cells (Figure 6A). As expected, cells exposed to UV irradiation without melanin pretreatment were strongly damaged, as indicated by the small number of living cells after treatment (about 20.0%). Differently, cells pretreated with melanin at 50 and 100 μg/mL showed strong protection against UV-induced damage (about 70.0% living cells), and the melanin-amyloid complex provided even higher protection (almost 100.0% living cells). Similar results have been observed by evaluation of the LDH activity in the cell medium of all experimental groups (Figure 6B). Indeed, while UV exposure promotes the release of LDH in the cell medium, which is associated with membrane damage (CTR+), cells preincubated with melanin showed a reduced LDH release that was stronger in the presence of the melanin-amyloid complex.

**Figure 6:**
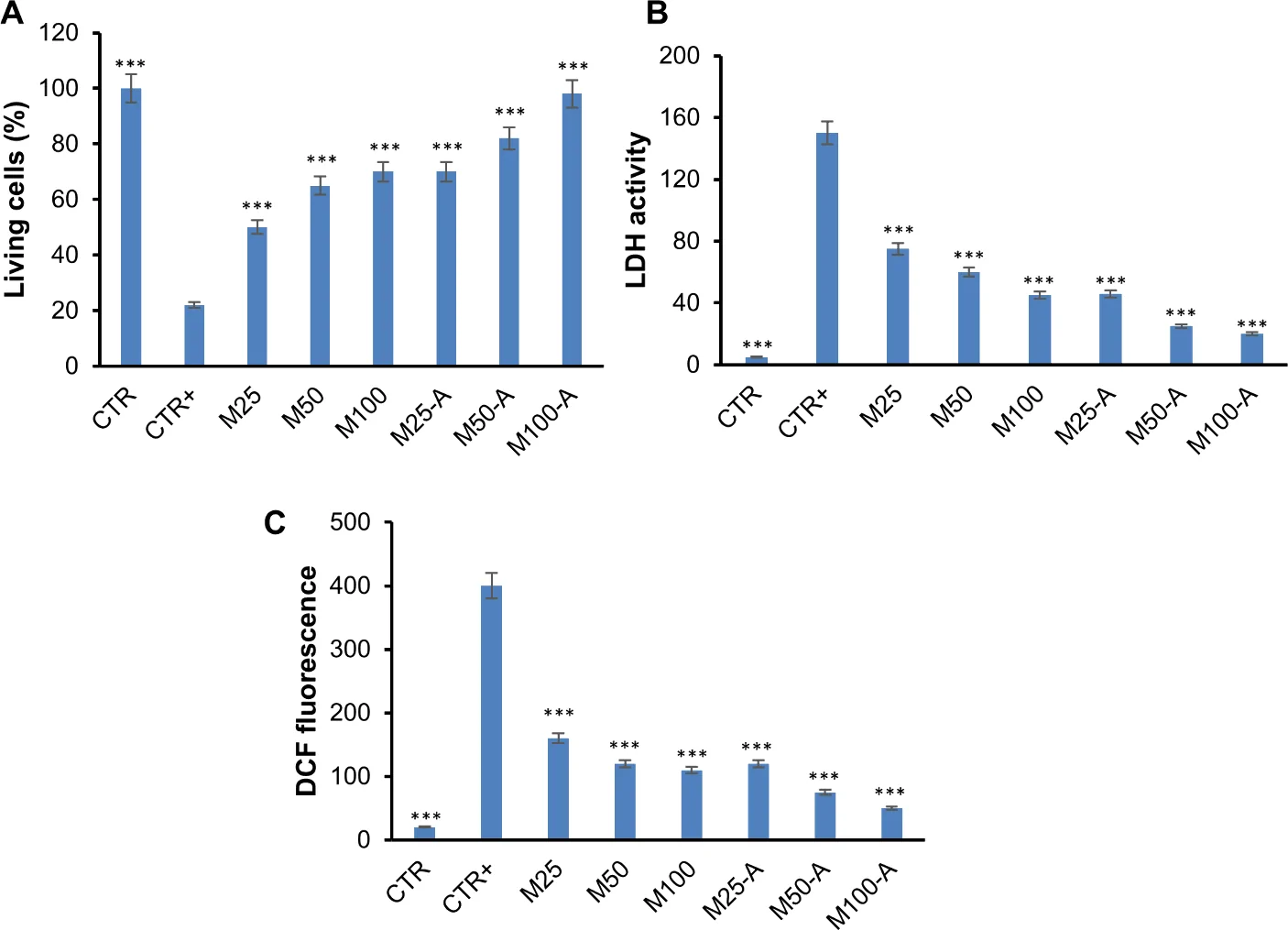
Protective effect of microbial melanin against UV radiation damage. (A) Trypan Blue assay, (B) LDH assay, and (C) DCFH-DA assay performed in HaCaT cells exposed to UV radiation after incubation with microbial melanin at 25, 50, and 100 μg/mL in the presence (M25-A, M50-A, and M100-A) and absence (M25, M50, and M100) of amyloid fibrils at a 1:1 concentration ratio. CTR: untreated cells; CTR+: cells exposed to UV irradiation. Data are expressed as the average number ± SD relative to untreated cells from triplicate wells from five separate experiments. Other experimental details are described in the Materials and Methods section. ***p < 0.05 versus CTR+.

These data suggest that microbial melanin is able to strongly protect human keratinocytes from UV-induced damage, and this protection is greatly enhanced by the presence of amyloid fibrils. A similar effect has been previously shown for synthetic melanin, which in the presence of an amyloid scaffold exhibited stronger photoprotection in HaCaT cells.^23^ In this study, the authors suggested that the incorporation of the amyloid scaffold alters the electronic properties of melanin, narrowing its bandgap and consequently broadening the capacity of the pigment to absorb and dissipate UV radiation. Since UV-induced damage is known to be associated with oxidative stress, we have also measured ROS production by the DCFH assay for the same experimental groups in order to identify the molecular basis of the cellular protection observed in the UV irradiation damage (Figure 6C).

As expected, UV exposure induced a strong increase in the DCF fluorescence, indicative of high ROS production. By contrast, cells preincubated for 5 h with melanin samples at different concentrations showed markedly reduced ROS levels (approximately 75.0% reduction), suggesting that microbial melanin was able to counteract UV-induced ROS production in human keratinocytes. Interestingly, this ability appeared to be higher in the presence of amyloid fibrils, thus explaining the higher photoprotection effect observed for the melanin-amyloid complex in HaCaT cells.

### 3.5 Effect of Melanin-Amyloid Complex as a Cytoprotective Agent against AGE-Induced Skin Damage

Advanced glycation end products (AGEs) are a heterogeneous group of compounds formed in the late stages of the glycation reaction, a non-enzymatic process originated by the reaction of reducing sugars such as glucose with free amino groups of biomolecules. AGE accumulation is linked to the development of numerous chronic diseases, including cardiovascular and neurodegenerative diseases, diabetes, and its complications, in which oxidative stress plays a crucial role. In addition, AGEs, besides contributing to skin damage and aging by altering skin structures and physiological functions, also act as pathogenic factors in skin complications associated with chronic metabolic diseases, such as diabetic ulcers, infections, and nonhealing wounds.^41–44^ The AGE-skin aging process is related to alterations in the physicochemical properties of dermal proteins, reduction in cell proliferation, and increased apoptosis and senescence induced by oxidative stress and inflammation.^45,46^ Several factors such as UV radiation and pollution exacerbate AGE accumulation. Keratinocytes, the predominant cell type in the epidermis, are involved in wound healing through their differentiation, proliferation, and migration, processes that are seriously affected by AGE accumulation following UV radiation exposure.^47,48^

In this respect, the protective effect of microbial melanin, in the presence and absence of amyloid fibrils, against AGE-induced toxicity was investigated in HaCaT cells, as this effect has never been tested before (Figure 7). In particular, the cytotoxicity was studied by the Trypan Blue assay in HaCaT cells pretreated for 5 h with 0, 25, 50, and 100 μg/mL microbial melanin and the melanin-amyloid complex (1:1 ratio) and exposed to 30 μM AGEs for 24 h (Figure 7A). As expected, AGEs promoted a strong reduction of living cells (around 60%), whereas in the presence of microbial melanin and the melanin-amyloid complex, low reduction was observed with 50 and 100 μg/mL showing the stronger effect in the presence of the melanin-amyloid complex. Similar results have been observed by evaluation of the LDH activity in the cell medium of all experimental groups (Figure 7B). Indeed, while AGEs promote the release of LDH in the cell medium, which is associated with cell toxicity (AGE), cells preincubated with melanin showed a reduced LDH release that was stronger in the presence of the melanin-amyloid complex. These data suggest that both microbial melanin and the melanin-amyloid complex are able to protect human keratinocytes from AGE-induced toxicity at both 50 and 100 μg/mL concentrations and that the presence of amyloid seems to enhance the microbial melanin protective capacity. To assess changes in cell morphology and cell number, the experimental groups were also examined by using phase contrast microscopy (Figure 7C). In accordance with the Trypan Blue assay, this analysis showed that cells exposed to AGEs for 24 h exhibited reductions in cell number and alterations in morphology, whereas those pretreated with microbial melanin and the melanin-amyloid complex showed no qualitative and quantitative changes.

**Figure 7:**
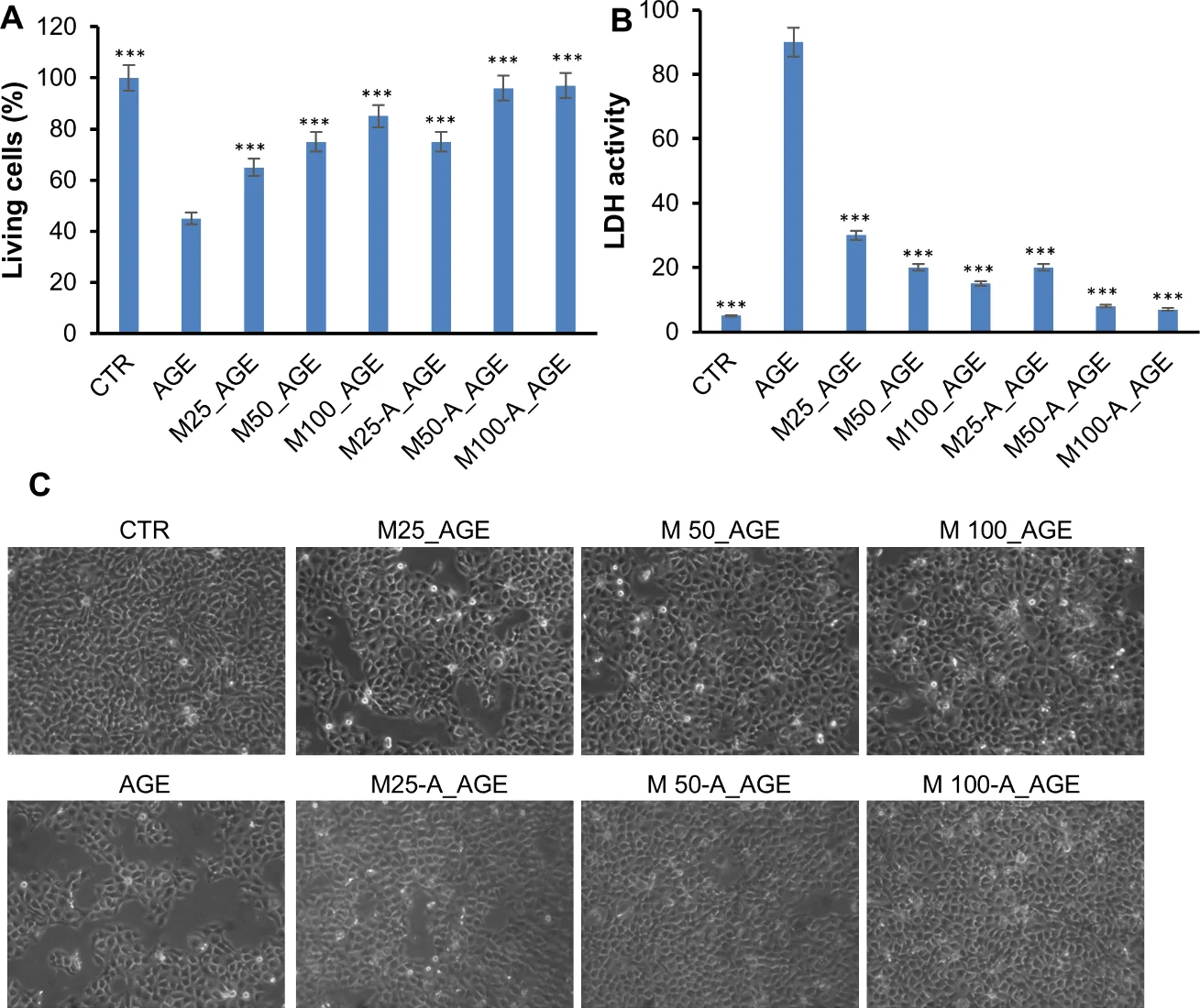
Cytoprotective effect of microbial melanin against AGE-induced toxicity. (A) Trypan Blue assay, (B) LDH assay, and (C) phase contrast microscopy images for HaCaT cells exposed to 30 μM AGEs after 5 h of preincubation with microbial melanin at 25, 50, and 100 μg/mL in the presence (M25-A_AGE, M50-A_AGE, and M100-A_AGE) and absence (M25_AGE, M50_AGE, and M100_AGE) of amyloid fibrils at a 1:1 ratio. CTR: untreated cells; AGE: cells exposed to 30 μM AGE. Scale bar: 50 μm. Data are expressed as the average number ± SD relative to untreated cells from triplicate wells from three separate experiments. Other experimental details are described in the Materials and Methods section. ***p < 0.05 versus cells treated with AGE alone.

AGE toxicity is known to be mediated by increased oxidative stress and inflammatory responses through their interaction with the RAGE receptor. Oxidative stress is a key mechanism by which AGEs exert cytotoxic effects primarily through increased ROS production, a pivotal event in activating molecular signaling pathways leading to inflammation and apoptosis.^49,50,51^ To identify the molecular basis of the protective effect of microbial melanin against AGE toxicity in human keratinocytes, we evaluated its effect on AGE-associated oxidative damage by measuring ROS production by DCFH assay (Figure 8).

**Figure 8:**
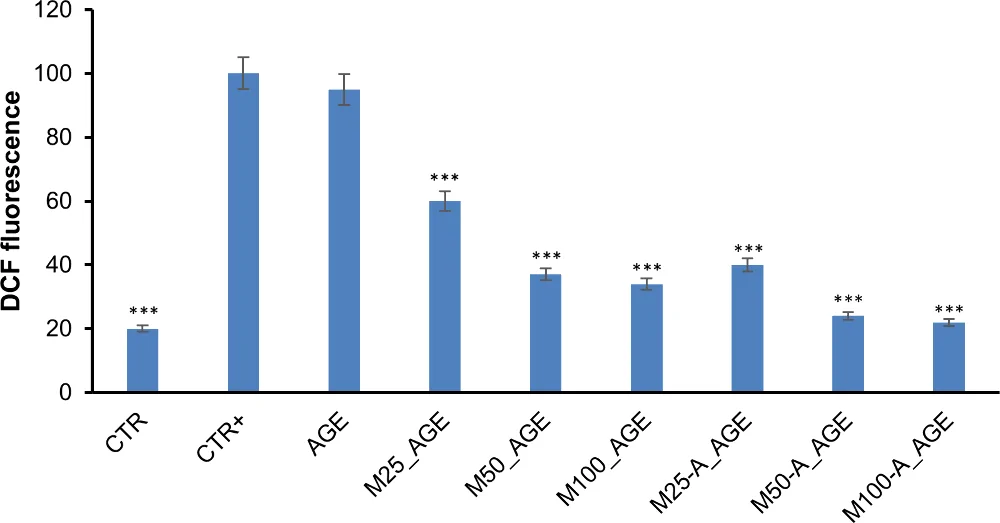
Protective effect of microbial melanin against oxidative stress induced by AGEs. DCFH-DA assay performed in HaCaT cells exposed to 30 μM AGE after 5 h of preincubation with microbial melanin at 25, 50, and 100 μg/mL in the presence (M25-A_AGE, M50-A_AGE, and M100-A_AGE) and absence (M25_AGE, M50_AGE, and M100_AGE) of amyloid fibrils at a 1:1 ratio. CTR: untreated cells; CTR+: cells exposed to 600 μM H_2_O_2_; AGE: cells exposed to 30 μM AGE. Data are expressed as the average number ± SD relative to untreated cells from triplicate wells from five separate experiments. Other experimental details are described in the Materials and Methods section. ***p < 0.05 versus cells treated with AGE alone.

As expected, AGE exposure promoted a strong increase in the DCF fluorescence, indicating high ROS production. By contrast, in the cells preincubated for 5 h with different melanin samples, the ROS levels were strongly reduced (around 60.0% reduction), suggesting that microbial melanin is able to counteract the AGE-induced ROS production in human keratinocytes. Interestingly, the protective effect observed for melanin was concentration-dependent, and the ability of microbial melanin to counteract AGE-induced oxidative damage appeared higher in the presence of amyloid fibrils. Indeed, in cells treated with the melanin-amyloid complex, ROS levels were comparable to those of untreated controls. These data showed, for the first time, that microbial melanin is able to protect human keratinocytes from AGE cytotoxicity by inhibiting oxidative stress. Similar to what was observed for microbial melanin in UV-induced damage, the protective effect was stronger in the presence of amyloid fibrils. Despite the promising results achieved using a single keratinocyte cell line, future studies should extend this research to primary human keratinocytes and attempt to establish a 3D skin model to validate these findings in a more complex and physiologically relevant cellular system. Nevertheless, these insights provide valuable knowledge, considering that both AGE toxicity and UV-induced damage are directly implicated in skin damage, suggesting a potential role for microbial melanin in protecting the skin against the aging process.

## 4 Conclusions

In summary, a functional characterization of microbial melanin by *Streptomyces nashvillensis* DSM 40314 was performed, and the pigment showed a strong antioxidant and photoprotective effect in human keratinocytes. In addition, we show for the first time that the presence of amyloid fibrils strongly improved the microbial melanin protective effects. These data suggest that the modulation of the melanin biological properties in the presence of amyloid fibrils could be mediated by a direct interaction between melanin functional groups and the amyloid structure. The augmented scavenger activity of microbial melanin in the presence of amyloid fibrils is likely responsible for the higher antioxidant activity and the stronger protection from UV irradiation damage observed in keratinocytes. Besides, for the first time, we show the ability of microbial melanin and the melanin-amyloid complex to protect human keratinocytes from AGE-related damage, a complex cellular pathway associated with skin damage and aging. In this respect, our study provides an interesting perspective for several innovative microbial melanin applications, also considering the role of amyloid fibrils in greatly enhancing their functional versatility. Moreover, this study adds knowledge about the role of functional amyloid in melanogenesis underlying the higher biological activity of eumelanin-like microbial melanin.

## Supplementary Material



## Notes

SEM images of the microbial melanin, HEWL fibrils, and melanin in the presence of HEWL amyloid fibrils at 1:1 and 1:2 concentration ratios (PDF)
