# Supplementary material for: Role of Amyloid Fibrils in Enhancing the Protective Properties of Microbial Melanin against Oxidative Stress, UV Damage, and AGE-Related Cytotoxicity in Human Keratinocytes
Source: ACS Omega. 2026 Aug 10;11(33):50028–39. doi: 10.1021/acsomega.6c05125 (PMC13520022; doi:10.1021/acsomega.6c05125)
Supplement: Supplementary file 1 [file ao6c05125_si_001.pdf]

Supporting information for

Role of amyloid fibrils in enhancing the protective properties of microbial melanin against oxidative stress, UV-damage and AGE-related cytotoxicity in human keratinocytes

Shana Perrella [1‡], Talayeh Kordjazi [2‡], Maria Liccardo [1], Loredana Mariniello [2], Ivana Sirangelo [1], Odile Francesca Restaino [2*], Clara Iannuzzi [1]*

1. Department of Precision Medicine, University of Campania “Luigi Vanvitelli”, Via L. De Crecchio 7, 80138 Naples, Italy.

2. Department of Chemical Sciences, University of Naples Federico II, Monte Sant’Angelo Campus, Via Cintia 4, 80126, Naples, Italy.

‡ Co-first authors

*Correspondence to: odilefrancesca.restaino@unina.it (O.F.R.)

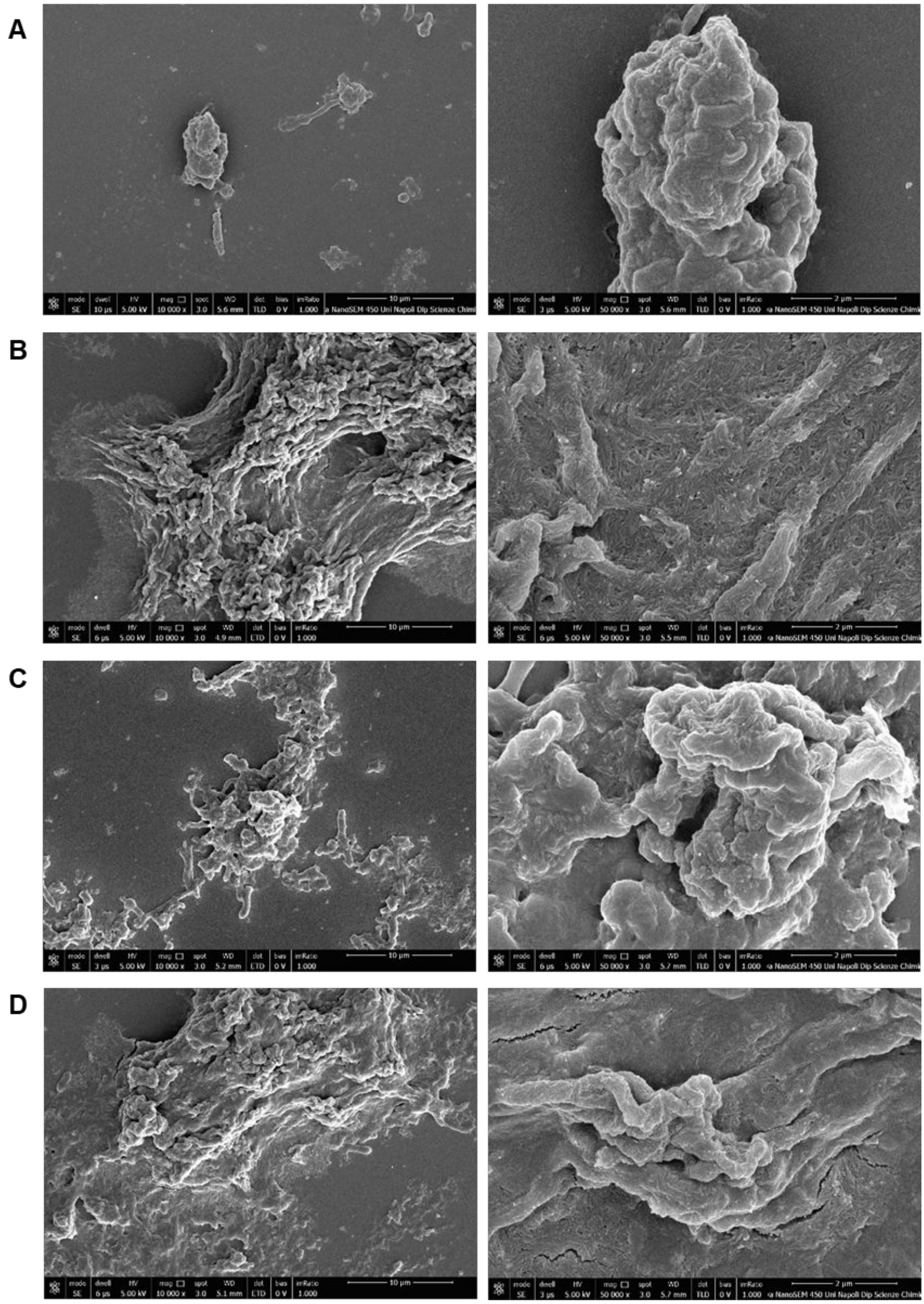

Figure S1. SEM images. The images are representative of microbial melanin (panels A), HEWL fibrils (panels B), and melanin in the presence of HEWL amyloid fibrils 1:1 (panels C) and 1:2 (panels D) concentration ratio. Melanin concentration was 0.25 mg/mL. Scale bar 10 µm (left panels) and 2 µm (right panels).
